# *Postmortem* Analyses Unveil the Poor Efficacy of Decontamination, Anti-Inflammatory and Immunosuppressive Therapies in Paraquat Human Intoxications

**DOI:** 10.1371/journal.pone.0007149

**Published:** 2009-09-25

**Authors:** Ricardo Jorge Dinis-Oliveira, Paula Guedes de Pinho, Liliana Santos, Helena Teixeira, Teresa Magalhães, Agostinho Santos, Maria de Lourdes Bastos, Fernando Remião, José Alberto Duarte, Félix Carvalho

**Affiliations:** 1 Faculty of Medicine, University of Porto, Porto, Portugal; 2 Department of Clinical Analysis and Public Health, Center of Research in Health Technologies (CITS)-IPSN-CESPU, CRL, Vila Nova de Famalicão, Portugal; 3 REQUIMTE, Department of Toxicology, Faculty of Pharmacy, University of Porto, Porto, Portugal; 4 Center of Forensic Sciences, Foundation for Science and Technology, Lisbon, Portugal; 5 Biomedical Sciences Institute Abel Salazar, University of Porto, Porto, Portugal; 6 National Institute of Legal Medicine I.P., Coimbra, Portugal; 7 CIAFEL, Faculty of Sport, University of Porto, Porto, Portugal; Sun Yat-Sen University, China

## Abstract

**Background:**

Fatalities resulting from paraquat (PQ) self-poisonings represent a major burden of this herbicide. Specific therapeutic approaches have been followed to interrupt its toxic pathway, namely decontamination measures to prevent PQ absorption and to increase its excretion from organism, as well as the administration of anti-inflammatory and immunosuppressive drugs. Until now, none of the *postmortem* studies resulting from human PQ poisonings have assessed the relationship of these therapeutic measures with PQ toxicokinetics and related histopathological lesions, these being the aims of the present study.

**Methodology/Principal Findings:**

For that purpose, during 2008, we collected human fluids and tissues from five forensic autopsies following fatal PQ poisonings. PQ levels were measured by gas chromatography-ion trap mass spectrometry. Structural inflammatory lesions were evaluated by histological and immunohistochemistry analysis. The samples of cardiac blood, urine, gastric and duodenal wall, liver, lung, kidney, heart and diaphragm, showed quantifiable levels of PQ even at 6 days post-intoxication. Structural analysis showed diffused necrotic areas, intense macrophage activation and leukocyte infiltration in all analyzed tissues. By immunohistochemistry it was possible to observe a strong nuclear factor kappa-B (NF-κB) activation and excessive collagen deposition.

**Conclusions/Significance:**

Considering the observed PQ levels in all analyzed tissues and the expressive inflammatory reaction that ultimately leads to fibrosis, we conclude that the therapeutic protocol usually performed needs to be reviewed, in order to increase the efficacy of PQ elimination from the body as well as to diminish the inflammatory process.

## Introduction

Paraquat (PQ) poisonings is by far one of the most clinically significant pesticide in terms of morbidity and mortality [1]. Acute PQ intoxications are mostly due to ingestion of the concentrated liquid herbicide formulations available in the market. The main target organ for PQ toxicity is the lung as a consequence of its accumulation against a concentration gradient, through the highly developed polyamine uptake system, and due to its capacity to generate huge amounts of pro-oxidant reactive species through a strenuous redox-cycle pathway [1]. Death occurs mostly as a consequence of alveolar epithelial cells (type I and II pneumocytes) and bronchiolar Clara cells disruption, haemorrhage, edema, hypoxemia, infiltration of inflammatory cells into the interstitial and alveolar spaces, proliferation of fibroblasts and excessive collagen deposition and as a consequence of a disseminated intravascular coagulation [1], [2], [3].

In view of the proposed mechanisms of PQ toxicity, several therapeutic measures have been taken, at different time points after intoxication, to hamper the toxic pathways [4]. In fact, over the past 50 years, strategies in the management of PQ poisonings have been directed towards: (i) modification of its toxicokinetics either by decreasing the absorption, by modifying the distribution or by enhancing its elimination from blood with forced dieresis and charcoal haemoperfusion (CHP), (ii) preventing the generation of reactive oxygen species (ROS), namely by the effective control of iron distribution by desferrioxamine, (iii) scavenging ROS though the maintenance of effective levels of antioxidants, such as vitamin E and *N*-acetylcysteine, (iv) repairing the ROS-induced lesions, particularly the maintenance of effective levels of glutathione by administrating *N*-acetylcysteine, and (v) reducing inflammation by dexamethasone, methylprednisolone, cyclophosphamide and *N*-acetylcysteine. A flowchart guide currently used in the management of poisoned patients was recently reviewed [1].

One of the major weaknesses of previous human *postmortem* studies related to fatal PQ poisonings, is the almost complete absence of correlation between the toxicokinetic of PQ and the respective histophatological lesions [1]. In addition, none of those studies assessed the correlation between human *postmortem* findings with the aggressive therapy that is currently performed, to evaluate its efficacy in terms of intended pharmacological effects. In attempt to provide new insights concerning the efficiency of strike points of the currently used therapeutic flowchart, we performed histological and toxicological analysis in fluids and tissues collected from five forensic autopsies carried out at the Portuguese North Branch of the National Institute of Legal Medicine (NB-NILM). Taking into account the high rates of mortality associated to PQ poisonings, we expect to provide reasoning for the inefficiency of the decontamination measures and of the anti-inflammatory and immunosuppressive therapies.

## Materials and Methods

### Ethics statement

All research was approved by the National Council of Ethics for the Life Sciences (CNECV). According to the current Portuguese Law for medico-legal autopsies, and following the ethical principles of Declaration of Helsinki, no informed written or oral consent of the victim family is required for scientific research in routinely collected tissues [5]. Therefore it is foreseen by the law to obtain samples beyond those establishing the cause of death.

### Chemicals

PQ (1,1′-dimethyl–4,4′-bipyridinium dichloride; molecular mass = 257.2 g/mol), ethyl paraquat dibromide (EPQ, 1,1′-diethyl-4,4′-bipyridinium dibromide; molecular mass = 374.11 g/mol), sodium borohydride (NaBH_4_), Mayer's haematoxylin solution, eosin Y disodium salt, Weigert's iron hematoxylin solution, van Gieson solution acid fuchsin, SIGMAFAST® Fast Red TR/Naphthol AS-MX Tablets and di-n-butylphthalate-polystyrene-xylene (DPX) mounting medium were obtained from Sigma (St. Louis, MO, U.S.A.). NF-κB p50 (NLS) rabbit polyclonal antibody (SC-114) and the secondary anti-immunoglobulin goat anti-rabbit IgG, F(ab′)2 conjugated with alkaline phosphatase (SC-3838), were obtained from Santa Cruz Biotechnology Inc., California, USA. Aquatex®, methanol (HPLC grade), Na_2_HPO4 (anhydrous), KH_2_PO4 (anhydrous), NaCl, KCl were all obtained from Merck (Darmstadt, Germany). All the reagents used were of analytical grade or from the highest available grade.

### Case reports and autopsies

A total of 5 human lethal intoxications with PQ (4 men and 1 women, aged 56–62) were included in this study. Intoxications were suspected upon arrival of patients at hospital emergency departments and subsequently confirmed by a spot test in urine sample using the alkali and sodium dithionite chromogenic reagent. Data concerning the amount of ingested PQ formulation and the performed therapeutic measures victim were registered. Deaths occurred in the interval of 9 hours to 6 days after the exposure. Cadavers were maintained at 2–3°C until autoptic examination. 1–3 days after death, autopsies were performed and samples of cardiac blood, urine, lung, liver, kidney, heart, diaphragm, duodenal and gastric wall were collected.

### Tissue processing for paraquat quantification

Lungs, kidney, liver, heart, diaphragm, duodenal and gastric wall samples were homogenized (1∶4 m/v, Ultra-Turrax® Homogenizer) in ice-cold deionized water. The homogenate was kept on ice, then centrifuged at 3000 *g*, 4°C, for 10 min. Aliquots of the resulting supernatants were stored (−80°C) for posterior PQ quantification. Cardiac blood and urine samples were directly subjected to PQ extraction procedures.

### Paraquat extraction from biological samples

PQ extraction from biological samples was performed according to de Almeida and Yonamine [6] with slight modifications. Briefly, an aliquot of 0.5 mL of each aqueous supernatant, urine and blood samples, 1.5 mL of phosphate buffered saline solution (pH 8.0) and 20 µL of EPQ solution (100 µg/mL) were pipetted into a 15-mL plastic tube with a screw cap. Ten milligrams of a sodium borohydride (NaBH_4_) were added to the solution. The reaction mixture was kept at 60°C for 10 min. For solid-phase extraction (SPE), the C18 (Bond Elut C18, bed weight 100 mg for 1 mL, Varian®) cartridge was preconditioned with 2 mL of methanol and 2 mL of phosphate buffered saline solution (pH 8.0). The sample solution was transferred into the cartridge and was further washed with 2 mL of deionized water. Afterwards, the elution of PQ was performed with 2 mL of methanol and the eluate was evaporated at room temperature under a gentle stream of nitrogen. The residue was reconstituted in 100 µL of methanol and 1 µL was injected into the gas chromatography-ion trap mass spectrometry (GC–IT-MS) apparatus.

### Gas chromatography-ion trap mass spectrometry conditions

GC–IT-MS analyses of PQ were performed using a Varian CP-3800 gas chromatographer (USA) equipped with a VARIAN Saturn 4000 mass selective detector (USA) and a Saturn GC/MS workstation software version 6.8. A chromatographic column, VF-5 ms (30 m×0.25 mm i.d. ×0.25 µm film thickness) from VARIAN, was used. The injector port was heated to 250°C and was operated in splitless mode. The carrier gas was helium (Gasin, Portugal), at 1.0 mL/min, constant flow. The oven temperature was 80°C (for 1 min), then increased 2°C/min until 270°C and held for 20 min. All mass spectra were acquired by electron impact (EI, 70 eV) in full scan mode. Ionization was maintained off during the first 2 min, to avoid solvent overloading. The ion-trap detector was set as follows: the transfer line, manifold and trap temperatures were 280, 50 and 180°C, respectively. The mass range was 50 to 600 *m/z*, with a scan rate of 6 scan/seconds. The emission current was 50 µA, and the electron multiplier was set in relative mode to autotune procedure. The maximum ionization time was 25000 μseconds, with an ionization storage level of 35 *m/z*. Chromatographic peaks' areas of PQ and EPQ were determined by re-constructed the FullScan chromatogram (FSC) using specific ions for each compound. A Selected Ion Monitoring Chromatogram (SIMC) was obtained. The ions selected for each compound were: *m*/*z* 134, 148, 192 (PQ) and *m*/*z* 148, 162, 220 (EPQ). The underlined ions were used for quantification.

### Tissue processing for structural analysis

Samples of lungs, kidneys, liver, heart, diaphragm, duodenal and gastric wall were submitted to the routine histological procedures for qualitative structural analysis. Briefly, cubic pieces were fixed [4% (v/v) buffered formaldehyde] by diffusion during 24 hours and subsequently dehydrated with graded ethanol and included in paraffin blocks. Benzene was used in the transition between dehydration and impregnation. Serial sections (4 µm) of the paraffin blocks were cut by a microtome and mounted on silane-coated slides.

### Staining procedures

The slides were dewaxed in xylene and hydrated through graded alcohols finishing in phosphate buffered saline solution prepared by dissolving Na_2_HPO4 (1.44 g), KH_2_PO4 (0.24 g), NaCl (8 g), KCl (0.2 g) and adjusting pH to 7.2. Deparaffinised sections were stained for haematoxylin-eosin and van Gieson protocols, and for immunohistochemistry NF-κB analysis, accordingly to previous described studies [7], [8]. Briefly, the haematoxylin-eosin staining was performed by immersion slides in Mayer's haematoxylin solution for 3–4 min followed by immersion in 1% eosin solution for 7 min, dehydration with graded alcohols through xylene, and mounting with DPX. The van Gieson staining [9], [10] was applied to evaluate collagen deposition. Slides were immersed in Weigert's haematoxylin solution for 20 minutes, washed in tap water for one minute, differentiated in acid ethanol (1% HCl in 70% alcohol) no more than 5 seconds, washed again in tap water for 5 minutes, rinsed in distilled water and immersed in van Gieson's stain for one hour. Finally, slides were rinsed quickly in distilled water and then in 100% ethanol, cleared and mounted in DPX. Collagen fibbers were evidenced by a red staining. To perform the NF-κB immunohistochemistry detection, NF-κB p50 rabbit polyclonal antibody was applied on deparaffinated liver sections and these samples were incubated at 37°C for 2 h in a humidified chamber. Samples were then incubated with a secondary anti-immunoglobulin goat anti-rabbit IgG, F(ab')2 conjugated with alkaline phosphatase, under the same conditions, for 1 h. SIGMAFAST® Fast Red TR/Naphthol AS-MX Tablets were used as substrate according to manufacturer's instructions. The sections were counterstained with Mayer's haematoxylin. The primary antibody was replaced by phosphate buffered saline solution for negative control sections. All stained sections were mounted on glass slides using Aquatex®. An optical photomicroscope Carl Zeiss - Axio Imager was used.

Considering the small sample size, the heterogeneity of PQ ingested dose and the variability of survival after intoxication among enrolled victims no statistical analysis was performed.

## Results

### Clinical, demographic and autoptic data

Accordingly to clinical reports, all the victims involved in this study had fever, a dry cough, and progressive dyspnoea. Regarding therapeutic measures, all victims were subjected to the same protocol, the differences among them being the plasma concentration of PQ and the survival period. Gastric lavage with activated charcoal and seven sessions of CHP (eight hours each) were performed. Further therapeutic measures were also carried out accordingly to the following protocol: (i) cyclophosphamide, 15 mg/Kg in 100 mL of a 5% dextrose solution, perfused over 60 minutes, once daily, after CHP, during the first two days of hospitalization; (ii) methylprednisolone, 15 mg/Kg in 200 ml of a 5% dextrose solution perfused over 60 minutes and repeated once daily for three consecutive days always after CHP; (iii) desferrioxamine, 100 mg/Kg in 500 ml of a 5% dextrose solution, in continuous perfusion, at 21 mL/h, during 24 hours, in one administration started after the first CHP session; (iv) vitamin-E, 300 mg *per os*, twice daily, after CHP; (v) *N*-acetylcysteine (NAC), was administered after the first CHP session in a dose of 150 mg/Kg in 500 mL of a 5% dextrose solution, perfused during 3 hours; subsequently, *N*-acetylcysteine was given at 300 mg/Kg in 500 mL of a 5% dextrose solution in continuous perfusion at 21 mL/h during 3 weeks. After 3 days, methylprednisolone was suspended, and the patients surviving received 5 mg of intravenous dexamethasone every 8 hours. In addition, each patient received prophylaxis for stress ulcer (omeprazol 40 mg I.V. twice daily) and for opportunistic infections (one daily tablet containing 800 mg cotrimoxazol and 160 mg of trimetoprim).

**Figure 1:**
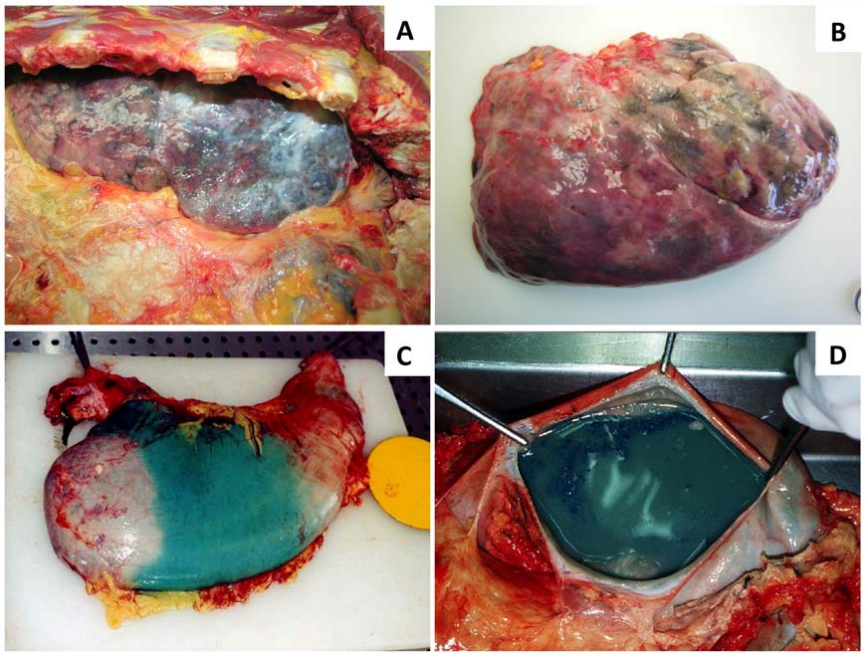
Autoptic macroscopic photodocumentation. A: lung fibrosis of case 5; B: lung edema of case 4; C and D: greenish blue colour appearance of gastrointestinal organs of case 1.

Table I shows data of the poisoning cases related to demography, ingested volume (mL) of formulation, body weight, organ weight, major macroscopic pathological findings and survival period after intoxication. The average age of the poisoned PQ victims was 58.6±2.2. Autoptic examination did not reveal any ulceration of the gastro-intestinal tract except in the third reported case, where gastritis was observed, but no relationship was established with PQ intoxication, since clinical reports documented previous pathology related to alcohol consumption and prolonged use of nonsteroidal anti-inflammatory drugs. The most prominent macroscopic findings were found in the lungs, which exhibited signs of fibrosis and increased weight due to edema (Figure 1A and 1B). Sub-pleural haemorrhages were also seen in the lungs. In all cases, kidneys and liver were significantly altered, with jaundice (yellow at both the surface and cut surface) and haemorrhage. In the case 1, a greenish blue colour was observed in the organs, corresponding to a rapid fatal PQ-poisoning (Figure 1C and 1D). There was no evidence of infection in all cases. Some variable associations could be inferred from the results presented in Table 1. As expected, the survival period was inversely related with the amount ingested. The lung weight seems to increase with the survival period whereas liver and heart weight looks like to decrease as survival period increases. No noticeable changes were observed for kidney's weight.

**Table 1:** Demographic data of fatal victims of paraquat poisonings and relevant clinical, autoptic and pathomorphological findings.

Autopsy case number	Age	Sex	Survival time after ingestion (days)	Paraquat dichloride intake (mL) of 20% w/v formulation	Weight (Kg)	Right/left lung weight (g)	Liver weight (g)	Right/left kidney weight (g)	Heart weight (g)	Main macroscopic pathological findings
1	58	M	9 h	50	85	750/720 (∼)	1900 (∼)	170/160 (∼)	600 (↑)	Pulmonary edema, haemorrhage and emphysema, greenish blue colour of organs
2	56	M	4	25	90	1700/1550 (↑)	1800 (∼)	230/280 (↑)	410 (∼)	Pulmonary and kidney edema and haemorrhage, and jaundice liver
3	59	M	2	40	82	950/780 (↑)	1500 (∼)	150/170 (∼)	340 (∼)	Pulmonary edema and fibrosis with emphysematous change, gastritis, kidney haemorrhage and jaundice liver
4	58	F	4	30	73	1100/950 (↑)	1230 (∼)	170/160 (∼)	270 (∼)	Pulmonary edema, emphysematous bullae with extensive haemorrhagic areas and jaundice liver
5	62	M	6	20	86	1200/1050 (↑)	1450 (∼)	180/170 (∼)	330 (∼)	Pulmonary edema and fibrosis, and jaundice and congested liver

It is also presented a comparative analysis with the reference values reported by de la Grandmaison and colleagues [52] for each organ weight: (∼), within normal range; (↑) increased weight.

### Histopathological analysis

**Figure 2:**
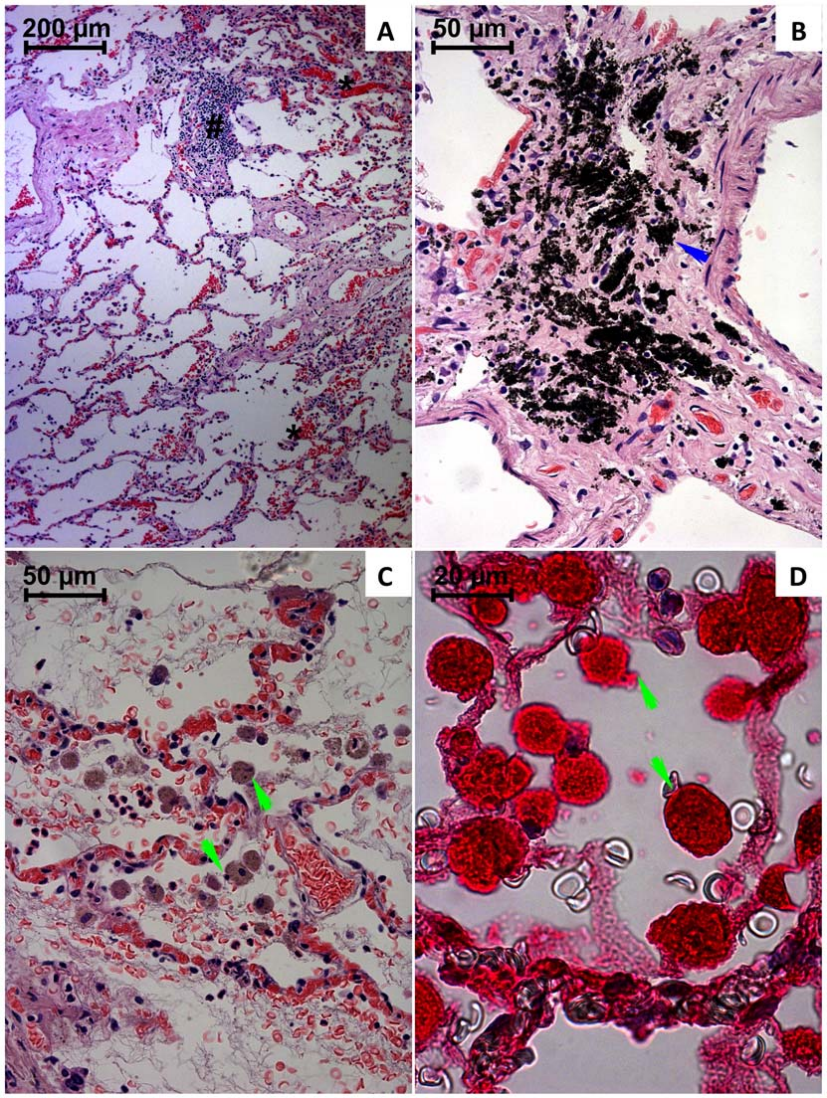
Light micrographs of lungs from paraquat human fatal poisonings stained with hematoxylin-eosin (A–C) and immunohistochemistry analysis for NF-κB (D). In A, it is shown an alveolar collapse with confluence of several alveoli, vascular congestion (*), enlargement of alveolar walls with leukocyte infiltration (#), alveolar hemorrhage and macrophage like-cells, and leukocytes within alveolar space. In B, it is depicted the accumulation of anthracotic pigment within large blood vessels walls (blue arrow). In C, it is observed vascular congestion, fibrin-like deposits within alveoli trapping red blood cells, leukocytes and macrophage-like cells (green arrow). In D, NF-κB activation is evident in macrophage-like cells (green arrow, D).

Major lung qualitative structural alterations are depicted in Figure 2. Lungs showed a marked alveolar collapse and enlargement of alveolar walls (Fig. 2A), apparently explained by the pronounced vascular congestion, interstitial edema and by collagen deposition, evidenced by van Gieson staining in 4 and 6 days-survival victims. Confluence of several alveoli was particularly evidenced. It was also observed signs of intralveolar diffuse coagulation, indicated by the presence of trapped red blood cells and leukocytes within fibrin-like deposits. Numerous macrophage-like cells (Fig. 2C) and infiltration of polymorphonuclear and mononuclear leukocytes were observed in all subjects in the alveolar space and walls. Thickening, rupture, or necrosis of the alveolar walls and desquamation of the pneumocytes was also particularly notorious. It was also noticed an extensive and dispersed deposition of anthracotic pigment within the wall of large blood vessels (Fig. 2B), in all observed cases.

**Figure 3:**
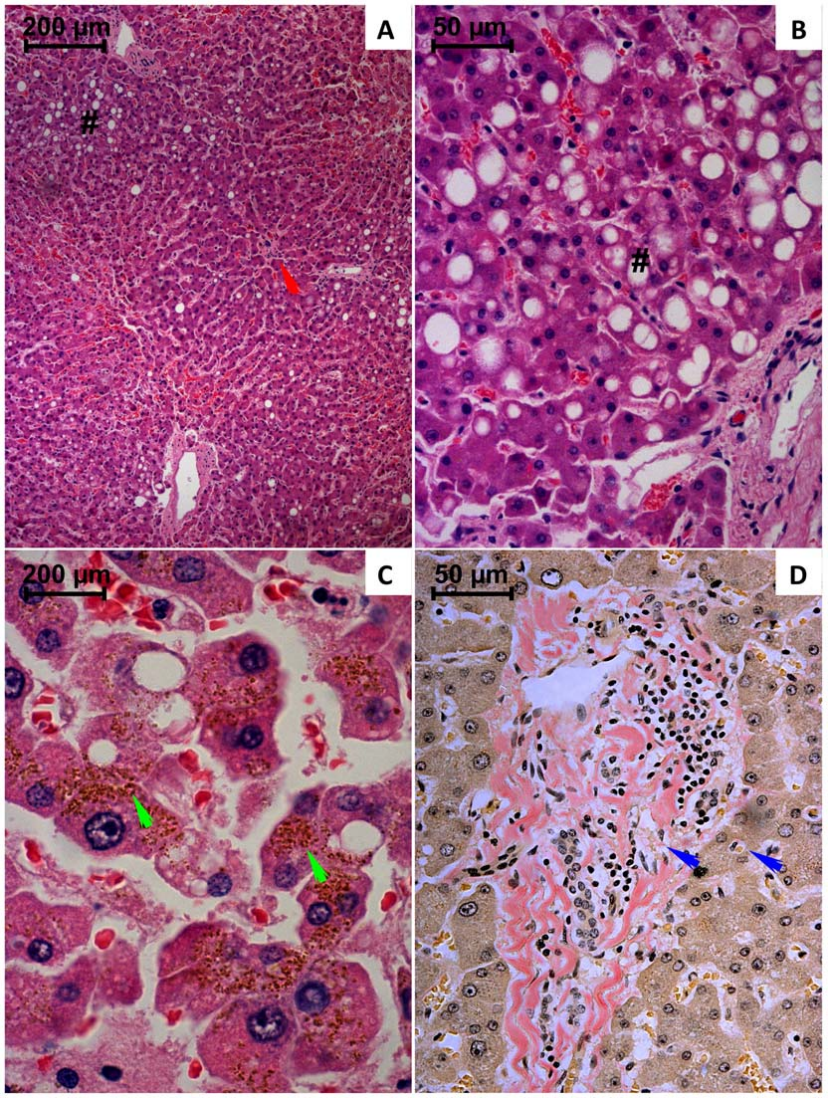
Light micrographs of liver from paraquat human fatal poisonings stained with hematoxylin-eosin (A–C) and van Gieson (D). In A, a marked enlargement of centrilobular sinusoids with erythrocytes trapped in the fibrinoid deposit; enlargement of large blood vessels walls with complete obstruction of a centrilobular vein (red arrow); macrovesicular vacuolization of periportal hepatocytes (#) it is also evident. In B, is shown macrovesicular (#) and microvesicular vacuolization in periportal hepatocytes. In C, it is observed near the centrilobular zone, extensive intracellular yellow-brownish deposits, cellular necrosis with numerous cell debris within enlarged sinusoidal spaces. In D, it is observed an intensive collagen deposition surrounding periportal and the sinusoidal spaces (blue arrow); it is also notorious the increase of cellular density in the periportal area, namely fibroblast-like cells and mononuclear leukocytes.

Major liver qualitative structural alterations are depicted in Figure 3. In the lobular structure, a marked enlargement of centrilobular sinusoids with erythrocytes trapped in the fibrinoid deposit and an increase of collagen staining surrounding sinusoids (Fig. 3D) was notorious, especially in victims with longer survival period. The intensive collagen deposition in the periportal and centrilobular areas resulted in stenosis of the major blood vessels. Infiltration with mononuclear cells and fibroblasts was evident in major blood vessel walls. Macrovesicular vacuolization near the periportal areas, and dispersed microvesicular vacuolization, was also observed (Fig. 3A and 3B). Histology also revealed extensive intracellular yellow-brownish deposits (Fig. 3C) and centrilobular necrosis.

**Figure 4:**
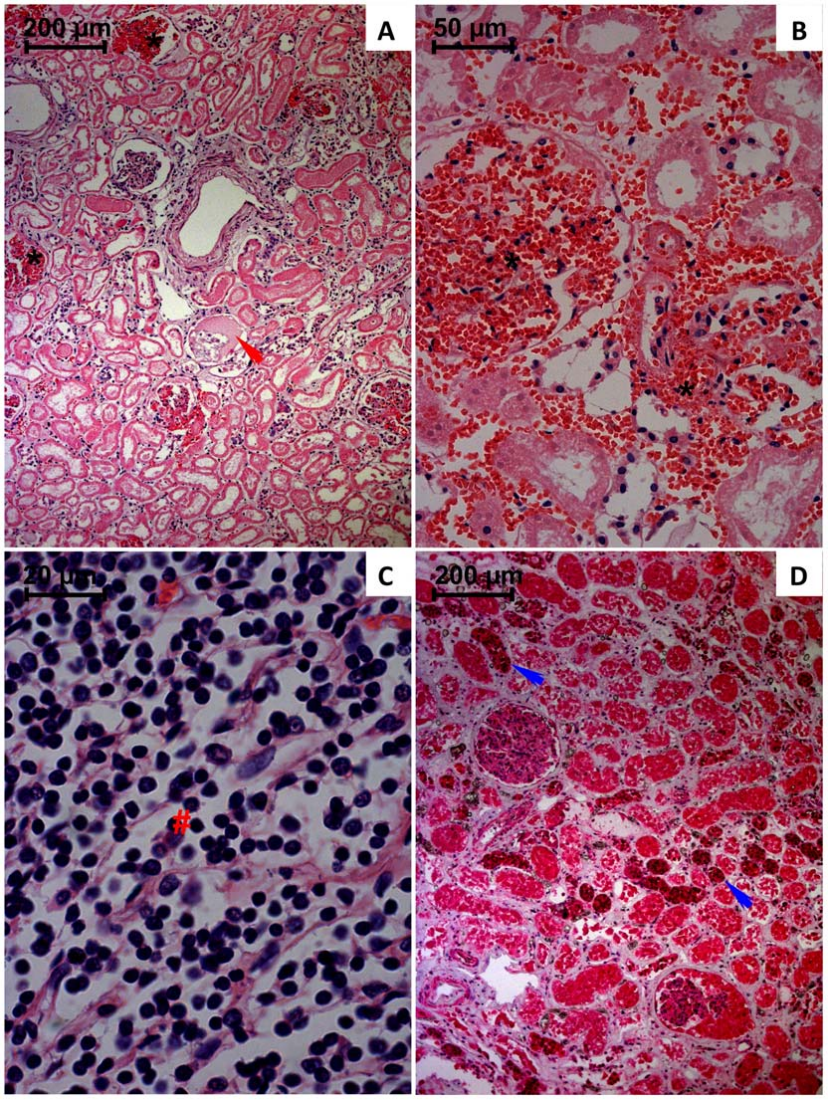
Light micrographs of kidneys from paraquat human fatal poisonings stained with hematoxylin-eosin (A–C) and immunohistochemistry analysis for NF-κB (D). In A and B it is observed marked vascular congestion within glomeruli and extraglomerular space (*); coagulation necrosis of glomeruli is also depicted (red arrow). Histology also revealed a widespread necrosis of tubular cells affecting more intensively the proximal convulated tubules. In C, pronounced density of mononuclear cells (#) within capillaries also infiltrating tissue. In D, NF-κB activation is generalized but particularly more intensive in the tubular distal convoluted tubules (blue arrow).

Major kidney qualitative structural alterations are depicted in Figure 4. Marked interstitial haemorrhage (Fig. 4A and 4B) and the collagen deposition in the interstitial space and surrounding large vessels, was observed. Histology also revealed necrosis of renal tubular cells, namely of the proximal tubules. The kidneys showed a thickening of the walls of blood vessels and of the parietal layer of Bowman's capsules, and global necrosis of glomeruli with its substitution by fibrinoid-like deposit. Sings of interstitial edema, vascular congestion and cell infiltration into the interstitium near the renal corpuscles was also evident (Fig. 4C).

**Figure 5:**
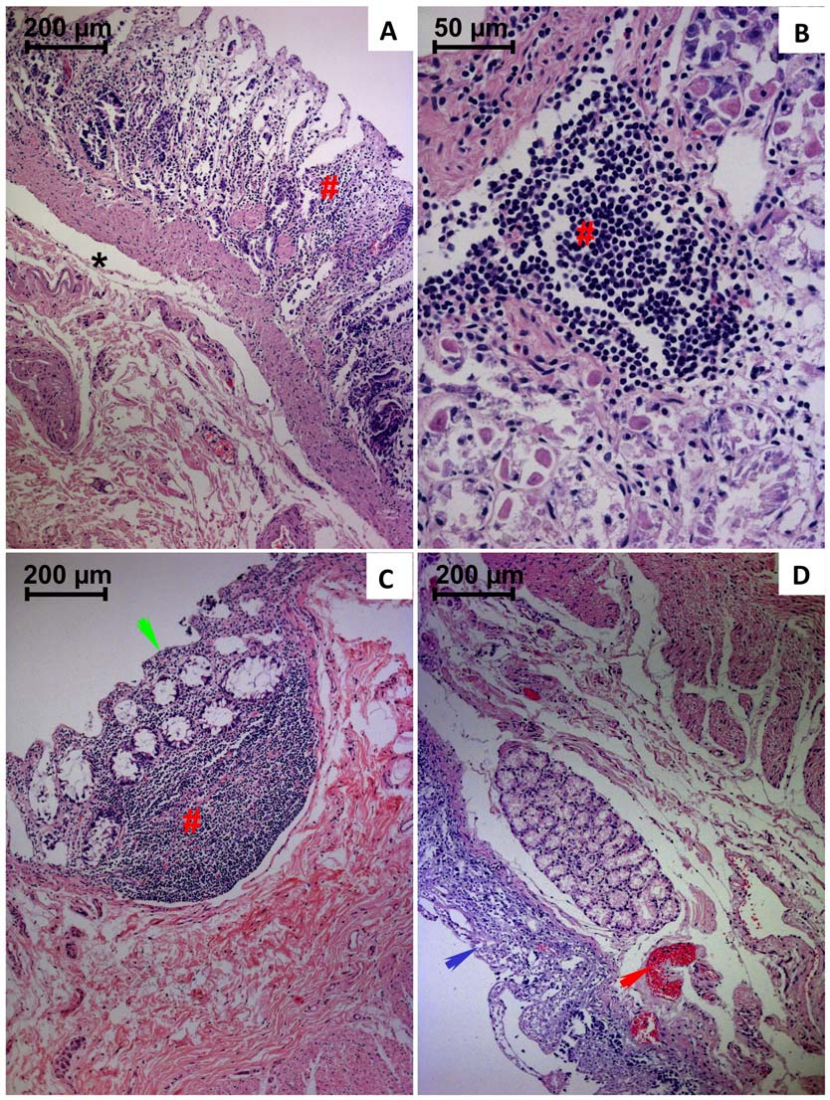
Light micrographs of gastric (A and B) and duodenal (C and D) wall from paraquat human fatal poisonings stained with hematoxylin-eosin. In A and B, it is observed necrosis of epithelial cells with desquamation and mucosal infiltration of mononuclear cells (#). There are also sings of submucosal edema (*). In C and D, epithelial necrosis affecting the villi and crypts with loss of mucosal villi structure, infiltration of mononuclear cells (green arrow), vascular congestion (red arrow), mucosal and submucosal edema, was particularly notorious.

Major gastric and duodenal wall structural alterations are depicted in Figure 5A–B and 5C–D, respectively. Loss of mucosal architecture, submucosal edema with necrosis and epithelial desquamation, was particularly observed. Confluent translucent areas were detected within villi and neighboring the crypts. Abnormal collagen deposition and mononuclear cells agglomerates were also notorious. Congestion and areas of haemorrhages were also noticed.

**Figure 6:**
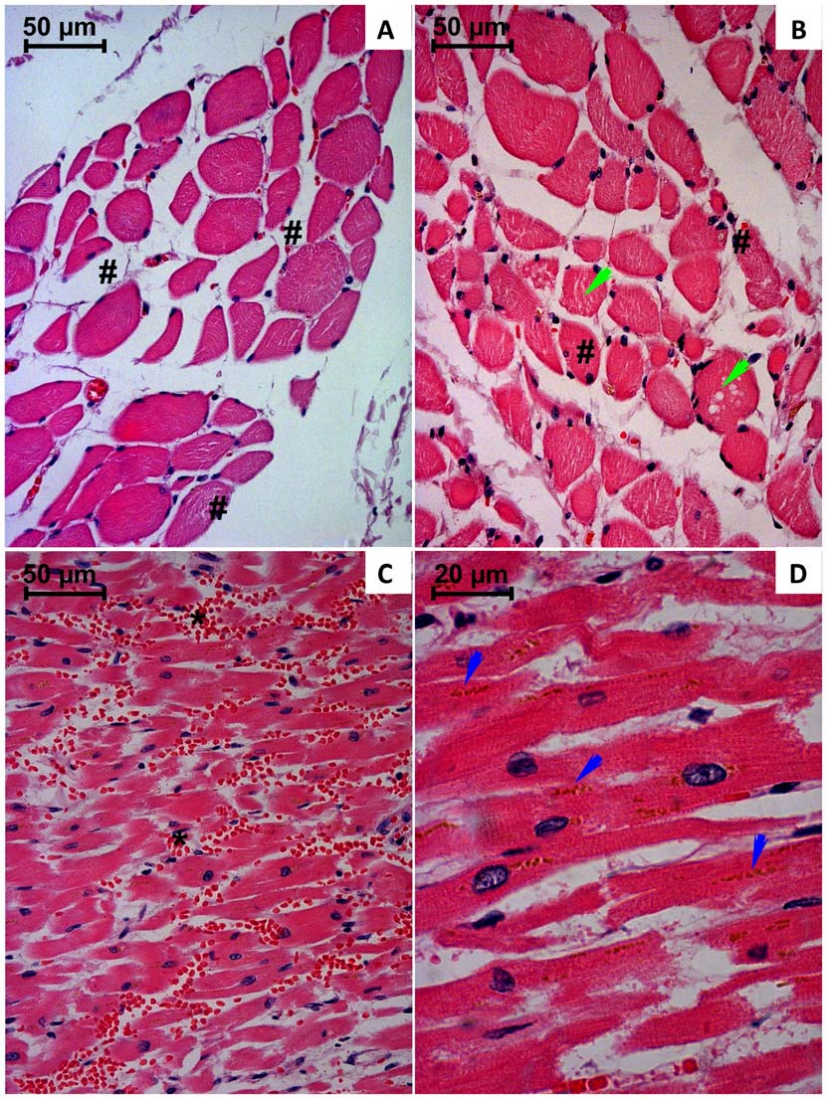
Light micrographs of diaphragm (A and B) and heart (C and D) from paraquat human fatal poisonings stained with hematoxylin-eosin. In A and B, it is observed signs of interstitial edema (#) and vacuolization (green arrow) within muscle fibers. In C and D, major microscopic alterations are related to vascular congestion (*), accumulation of yellow-brownish pigments (blue arrow) and fibrin-like deposits within interstitial spaces.

Major diaphragm and heart qualitative structural alterations are depicted in Figure 6. The heart examination revealed areas of interstitial edema, localized hemorrhagic infiltration and numerous marginalized leukocytes within capillaries. It was also observed intracellular yellow-brownish deposits near the perinuclear areas, vascular congestion and necrotic focus affecting a small number of cells. Regarding to the diaphragm, it was also observed vascular congestion, interstitial edema, perinuclear yellow-brownish deposits, an intensive sarcoplasmatic vacuolization and numerous fibers with central nucleus.

### Immunohistochemistry NF-κB analysis

Immunohistochemistry analysis was performed in order to assess whether the activation of the inflammatory transcriptional factor, NF-κB, was counteracted by the applied anti-inflammatory and immunosuppressive therapies. All analyzed tissues showed positive staining for activated NF-κB, affecting the majority of the cells. In the lungs a strong activation was particularly notorious in the macrophage-like cells (Fig. 2D). Tubular distal convoluted cells (Fig. 4D) revealed the most intensive staining. NF-κB immunohistochemistry staining was more evident within the crypt cells of gastric and duodenal mucosa. Concerning remaining observed tissues, the NF-κB positive staining did not show any particular cellular preference, the organ being entirely affected (results not shown).

### Paraquat quantification

Paraquat concentration in organs (μg/g of tissue), and urine and blood (μg/mL) *postmortem* samples are presented in Table 2 as independent results for each reported case. Due to heterogeneity of the ingested dose, vomit volume and survival period, no average concentrations were calculated. Nevertheless, it was possible to infer some valuable results: (i) lung was the organ with the highest concentration within *postmortem* cases of all analyzed samples except in the first case, where urine showed the highest concentration, which is in accordance with the rapid fatal intoxication; (ii) kidney revealed the ensuing highest concentration; (iii) heart exhibited the lowest PQ concentration; (iv) blood is not always a good matrix to perform PQ quantitative analysis and (v) PQ is still present in all tested organs, even 6 days after poisoning.

**Table 2:** Paraquat concentration in organs (μg/g of tissue), urine and blood (μg/mL) collected *postmortem*.

	Concentration (μg/g of tissue or μg/mL for urine and blood)				
**Organ/Fluid**	Case 1	Case 2	Case 3	Case 4	Case 5
Lung	11.856	0.660	5.095	2.231	0.500
Liver	4.355	0.340	0.973	0.879	0.100
Kidney	5.662	0.575	1.044	1.145	0.998
Heart	0.023	0.001	0.003	0.007	Not detected
Duodenal wall	3.100	0.260	2.320	0.168	2.830
Gastric wall	1.234	0.190	0.200	0.094	0.160
Diaphragm	0.305	0.016	0.130	0.131	0.010
Urine	13.539	0.078	0.592	0.177	0.500
*Postmortem* cardiac blood	9.500	Not detected	0.290	0.090	Not detected

## Discussion

According to the most recent casuistic studies, PQ is responsible for thousands of fatal poisonings due to pesticides exposures [11], [12], [13], [14], [15], [16]. In a recent study performed in Portugal, it was estimated that 20 to 30% of all forensic *postmortem* analysis of pesticides, resulted from PQ poisonings [17]. PQ was banned by a European Union court for not meeting health standards on 11^th^ July 2007, annulling therefore the approval of 2003. Nevertheless, during the following 12 months after decision it was possible to sell PQ present in stocks and to use it until December 2008. In spite of these new regulations, during 2008 in Portugal, PQ still represented 10% of all fatal cases. In all cases, suicide intent by ingestion was the only reported route of intoxication used, sustaining the consensual thought that it is safe when properly used. The high mortality is mainly due to the lack of effective treatments. In fact, much is still unknown concerning PQ toxicity mechanisms and even less about the adequate treatment measures. In attempt to understand the fragilities of the current treatments for PQ poisonings, we studied the first 5 human victims of PQ poisonings during 2008 in the NB-NILM. The results obtained unveil the reasons why the followed therapy did not achieve its biological/survival objectives. In fact, neither the elimination therapies nor the treatments directed to counteract the inflammatory processes showed to be efficacious in reverting PQ-related pathophysiological alterations. *Postmortem* concentration of PQ in different tissues reveals that applied treatments did not prevent lethal tissue accumulation. Furthermore, histophatological analysis showed a severe incidence of important structural changes especially concerning the inflammatory reaction.

The survival period of the victims enrolled in this study varied between 9 hours and 6 days, representing fatal acute intoxications. The severity of the histological, immunohistochemistry and toxicokinetic findings reflects the ingested dose, although the real amounts absorbed are highly dependent on emesis and/or gastric lavage. Lungs were abnormally heavy as shown in Table 1, congested and oedematous, filling and holding the shape of the thoracic cavity. Macroscopic signs of fibrosis were observed in the fifth case, which is in accordance to the survival period. Microscopically, all exhibited positive staining to van Gieson technique, reflecting fibrosis, but without enough extension to contribute significantly for the fatal outcome. It appears that the hypercellular “proliferative” phase, with loss of the alveolar structures due to intra-alveolar and interstitial collagen deposition was not the major mechanism of death of these victims. According to literature, reported cases of moderate intoxications with subsequent longer survival periods, show rather different findings at autopsy [18]. The changes in the mucous membranes of the oropharynx are eventually resolved and the liver and kidneys are usually of normal appearance, the dramatic changes being found in the lungs, which denote the well known-classical picture of PQ poisoning. On gross examination, the lungs are usually of reduced size, with a solid appearance due to fibrosis and presenting dark grey colour. By microscopic analysis, it is usually observed a grossly abnormal tissue with abundant fibrosis, often virtually obliterating the alveoli. Many plump fibroblasts are to be seen in alveolar walls and alveolar spaces [18]. In general, the longer the survival time, the more marked is the proliferation of fibroblasts in the alveoli, the more airless the lung tissue is, and less inflammation is usually observed [18]. This well known pattern was not evident in the fatal PQ intoxication cases included in this study since all deaths were observed within hours to few days, not allowing enough time for the massive synthesis and deposition of collagen to occur. Pronounced vascular congestion, signs of intralveolar diffuse coagulation, suggested by the presence of trapped red blood cells and leukocytes within fibrin-like deposits, numerous macrophage-like cells showing strong NF-κB activation and infiltration of polymorphonuclear and mononuclear leukocytes were observed in the alveolar space and walls from all subjects. These findings suggest that the immunosuppressive and anti-inflammatory drugs are not efficiently reverting PQ-induced lung toxic effects. In addition, it seems to be obvious that an antithrombotic drug should be included in the arsenal to be used in the management of PQ poisonings to avoid intravascular and intralveolar coagulation. Another interesting result, coming from histology, relates to the deposition of anthracotic pigment within the walls of large vessels of lungs and in the cytoplasm of alveolar macrophage-like cells. This phenomenon was not previously described. All intoxication cases were coming from semi-urban areas and none of the victims exhibited previous history of tobacco smoking practice. We hypothesise that the carbon particles deposit is the consequence of the release from charcoal cartridges used in CHP, which may represent another secondary effect of this therapeutic measure. The goal of extracorporeal elimination procedures is to remove PQ from the circulation and prevent its uptake by pneumocytes and Clara cells. The only method that has been shown to effectively enhance the extracorporeal elimination of PQ is indeed CHP. Most toxicologists currently recommend rapid initiation of CHP to lower plasma PQ levels and to limit pulmonary and other organs uptake of PQ. Analyzing 105 patients, who had swallowed one to three mouthfuls of PQ solution (24.5% w/v), Hong *et al.* [19] concluded that adequate CHP appears to be an indispensable treatment for patients with acute PQ poisoning. Okonek *et al.* [20], [21] proposed that “continuous” (repeated) CHP should be performed. The victims included in our study were submitted to seven sessions of 8 hours each during 4 days, when the survival period allowed it. Although there are considerable evidences of CHP efficacy in the reversion of the fatal outcome resulting from PQ poisonings, the usefulness of this therapy has been the subject of significant controversy with several evidences published in the literature showing a lack of clinical benefit in numerous cases [22], [23]. Our *postmortem* study corroborates that CHP was not capable to completely remove PQ from organism, even after the 6 days of survival that permits seven sessions of CHP. In our opinion, unless the procedure is begun at an early stage, when PQ is concentrated in the central compartment, a poor total body PQ clearence by extracorporeal techniques will result. This can be explained by the extensive PQ tissue distribution, as it was observed in this study, and as consequence of its slow redistribution back into the circulation following termination of the extracorporeal removing procedure [24]. In accordance, a rise of PQ concentrations, for several hours following completion of CHP, may ensue, supporting the thought that even in the presence of continuous CHP, the efficacy is not clear.

Concerning to structural alterations of the kidney, extensive areas of infiltration of inflammatory cells, necrosis, haemorrhage and jaundice were observed. Immunohistochemistry analysis revealed marked NF-κB activation of distal convoluted cells (Fig. 4D). The pronounced activation of NF-κB at the distal tubules comparatively to proximal ones could be explained by the almost complete absence of proximal tubules as result of extensive necrosis. Indeed, the proximal tubules seem to be more affected as consequence of PQ poisoning, which supports the lower capability to activate NF-κB. Beebeejaun and colleagues [25] also found proximal renal tubular necrosis by histopathological examination of a fatal case of PQ poisoning. According to previous reported studies, PQ is mainly eliminated by tubular filtration and active tubular secretion in humans [26], tubular reabsorption being minimal [25]. In humans, over 90% is excreted unchanged within 12 to 24 hours after ingestion, if renal function remains normal [27]. Ingestion of large doses of PQ causes tubular necrosis with a rapid decrease in the GFR and tubular secretion, and the consequent increase of the elimination half-life [26], [28]. After lung, kidney was the organ that evidenced the highest PQ concentration, which supports the significance of this route for PQ elimination.

In the liver, a marked enlargement of centrilobular sinusoids was observed, and an increase of collagen staining surrounding sinusoids was also notorious (Fig. 3D). An obvious stenosis of major liver blood vessels was observed, suggesting compromised perfusion of this major metabolic organ. Liver histology also evidenced ample deposition of yellow-brownish pigments (Fig. 3C). Although it was not possible to accurately identify the reason for the appearance of these pigments, this finding could be related to cholestasis, since livers showed to be jaundiced in all victims. In accordance, the analysis of 13 patients with hepatic injury associated with PQ poisoning, Mullick *et al*. [29] showed damage of the intrahepatic bile excretory pathways in ten of these patients. The authors described two phases in PQ hepatotoxicity, the first being due to accumulation of PQ and manifested by hepatocellular injury, and the second characterized by cholangiocellular and cholestatic damage related to the excretion of PQ into the bile or by absorption via enterohepatic circulation, with subsequent elimination into bile. Takegoshi *et al.* [30] also showed the liver damage involvement in acute PQ poisoning with intrahepatic cholestasis and jaundice, and mild hepatocellular necrosis. In addition, these authors also observed injury of bile excretory pathways in liver biopsies. Ultrastructurally, dilatation of bile canaliculi with decrease of microvilli and thickening of pericanalicular ectoplasm was found in the hepatocytes [30]. Other authors also showed that intrahepatic cholestasis in PQ poisoning in humans is secondary to extensive bile duct injury [31], [32]. These findings corroborate our results and suggest that bile secretory apparatus in the hepatocytes as well as biliary epithelial cells could be a target for PQ. The biliary route seems to represent an important pathway for PQ excretion due to the strong expression of P-glycoprotein (P-gp) at the canalicular membrane of hepatocytes [33], [34] and PQ has been recovered in the bile *postmortem* samples [35]. In fact, P-gp was recently demonstrated to be actively involved in the transport of PQ [36]. Apart from excretion role of the biliary tract, this fact also suggests that enterohepatic recirculation should be considered to occur in humans. Liver was shown to be the third organ to accumulate PQ. This is probably due to continuous absorption of PQ from the intestinal tract, and may reflect the above mentioned enterohepatic recirculation, or a relative inefficiency of the administration of activated charcoal in the gastric lavage procedure, to decrease absorption of PQ by the intestinal tract. The presence of PQ in the gastric and duodenal mucosa wall in all cases is in agreement with these postulations. One should be clearly aware that absorption occurs primarily in the small intestine (poorly from the stomach) and is estimated to be 1–5% in humans over a 1–6 hours period [27], [37], [38].

Regarding to the diaphragm specimens examined in our study all showed various degrees of degeneration, including swelling, changes of cross striation, vascular congestion, interstitial edema, perinuclear yellow-brownish deposits, an intensive sarcoplasmatic vacuolization and numerous fibers with central nucleus. The heart muscle evidenced similar pattern of injury. Histological examination revealed areas of interstitial edema, localized hemorrhagic infiltration and numerous marginalized leukocytes within capillaries. It was also observed intracellular yellow-brownish deposits near the perinuclear areas, vascular congestion and necrotic focus affecting a small number of cells supporting the thought that this organ was not as well protected by the performed therapy. Myopathy associated with PQ poisoning was reported, for the first time, by Saunders and coworkers in 1985 [39]. Koppel and colleagues [40] subsequently reported that extensive myonecrosis was observed in a specimen of *postmortem* intercostal muscle of a 52-year-old woman who had ingested an unknown dose of PQ and died on the 11^th^ day after ingestion. Vyver *et al.* [41] described a case of a patient that died 5 days after ingestion of PQ, whose PQ levels were high in the skeletal muscle and an increase of creatinine kinase levels in blood appeared on the fourth day after hospital admission. In rat experiments carried out by Sharp *et al.* [42] and by Rose *et al.* [43], a short time after the oral PQ administration, concentrations in skeletal muscle, were lower than those found in lung, kidney and liver. In addition, Sharp *et al.* [42] reported that the subsequent half-life of PQ was longest (4–5 days) in muscle, though the initial half-life of PQ in both plasma and other tissues was extremely short (20–30 min). They also reported that, in rats, the decline of PQ levels was slowest in muscle, and that muscle represented a major residual pool of PQ. In the present study we could not assess this depot behavior of muscle. PQ was detected in diaphragm in all cases and in the heart in four cases, but since these cases represent acute or subacute fatal intoxications, there was not enough time to verify the decrease of PQ accumulation in the lung, liver and kidney, and the maintenance, due to higher half-life, of PQ levels in muscular tissue. More recently, degeneration of skeletal muscle, mainly of the rectus abdominis, psoas major and diaphragm were also reported in fatal human PQ poisonings [44]. In previous preclinical studies performed in rats no relevant structural changes or differences in the collagen deposition in heart muscle were noticed 30 days after PQ intoxication [7]. With a different experimental design, Noguchi *et al.* [45] observed severe edema, congestion and haemorrhage and disfunction in the heart of rats that had died shortly after administration of a large amount of PQ (364 mg/kg). These authors suggested that a rapid accumulation of PQ into the heart in the early stages of exposure may play an important role in acute death. Toxic myocarditis was also reported by other authors in humans as resulting from PQ intoxication [46], [47], [48]. Povoa *et al.* [49] reported that cardiac toxicity due to PQ is frequent (40%). The clinical picture of this involvement had a wide spectrum, ranging from minimal changes in the electrocardiogram to acute and extensive myocardial necrosis.

The well known caustic effect of PQ has been responsible for ulcerated lesions in the lips, tongue, oropharynx, esophagus, stomach and trachea mucosa especially in severe intoxications [50]. Multiple pearly ulcerations in the oral cavity, necrotic ulcers in the trachea and entire bronchial tree, and hyperemic tracheal mucosa with abundant material of purulent appearance that permitted visualization of a break in the continuity of the posterior tracheal wall, were also observed by bronchoscopy in PQ intoxicated patients [51]. However, none of the victims enrolled in the herein described study evidenced such lesions. In the third case, gastritis was observed, but was associated with a previous pathology related to alcohol consumption and prolonged use of nonsteroidal anti-inflammatory drugs. Accordingly to circumstantial findings, Gramoxone® was the formulation ingested by all victims. This formulation possesses safeguard measures, namely a blue-greenish dye, emetic and odour. Accordingly, the observed greenish blue colour of organs registered in the case 1 is related to PQ formulation.

In conclusion, this study demonstrates that the currently used therapeutic flowchart needs to be refined, since neither the accumulation, nor the injuries related to PQ exposure seem to be effectively reverted. The use of pharmacological treatments to prevent PQ toxicity in undergoing preclinical and clinical trials, if successful, will certainly contribute to lower the human morbidity and mortality related to this herbicide.
